# Dose-response relationship between 15 weeks of running and aerobic fitness: a retrospective analysis based on the fun running program

**DOI:** 10.1186/s12889-024-18484-z

**Published:** 2024-04-12

**Authors:** Zhixuan Tao, Xugui Sun, Jun Sun, Ergang Zhu

**Affiliations:** Department of Public Foundation, Wannan Medical College, 241000 Wuhu, China

**Keywords:** Dose-response relationship, Fun running, Aerobic fitness, College students, Post-epidemic era

## Abstract

**Background:**

Students’ physical fitness, particularly aerobic fitness, has seriously declined during the COVID-19 epidemic. However, in the post-epidemic era, there are few studies on the methods of improving aerobic fitness. Understanding the dose-response relationship between physical activity and aerobic fitness is crucial for developing effective exercise prescriptions.

**Method:**

This retrospective study reviewed the Fun Running program at Wannan Medical College in China. We conducted a pre-post study design to analyze the impact of 15 weeks of Fun Running training on aerobic fitness. Middle and long-distance running pace (MLDR-P) was used as the primary indicator of aerobic fitness. A paired sample T-test was used to analyze the differences between the two MLDR-Ps. Pearson’s correlation was used to examine the correlation between variables. Multiple linear regression was used to determine the extent to which Fun Running components explain the variance in MLDR-P.

**Results:**

A total of 3244 college students participated in this study. 15 weeks of Fun Running training can significantly improve the MLDR-P in both females (*P* < 0.001, ES = 0.68) and males (*P* < 0.001, ES = 0.72). The MLDR-P was significantly correlated with Fun Running (R^2^ = 0.95, *p* < 0.05, for females; R^2^ = 0.96, *p* < 0.05, for males). The component that had the greatest impact on MLDR-P was pace (β = 1.39, for females; β = 1.09, for males), followed by distance (β = 0.49, for females; β = 0.15, for males), and last frequency (β = -0.03, for all).

**Conclusion:**

This study fills the gap in research on the dose-response relationship between running and aerobic fitness among college students in the post-epidemic era. The results show that 15 weeks of Fun Running training can significantly improve aerobic fitness. Examination of the dose-response relationship between Fun Running and MLDR-P provides practitioners with valuable insights into prescribing aerobic fitness training, allowing them to develop more effective training programs. Future research should focus on how to implement a hierarchical Fun Running program effectively.

## Background

In the wake of the COVID-19 pandemic, students across the globe have experienced a considerable decrease in their physical activities [1–3]. Additionally, the widespread adoption of online courses has further exacerbated the sedentary behavior of students during the pandemic [4, 5]. Consequently, there has been a significant decline in the physical fitness of students [6, 7], particularly in aerobic fitness [8]. Therefore, it is very urgent and important to conduct in-depth research on how to effectively enhance the aerobic fitness of students in the post-epidemic era.

Aerobic fitness, serving as a critical indicator for assessing an individual’s cardiopulmonary health [9], has been confirmed by numerous studies to be associated with the risk of cardiovascular diseases [10–13]. Specifically, a high level of aerobic fitness is closely linked to a lower risk of cardiovascular diseases [10, 11]. Conversely, individuals with inadequate cardiorespiratory function often face a higher overall mortality rate and an increased risk of cardiovascular diseases [12, 13]. Therefore, enhancing aerobic fitness is not only crucial for an individual’s overall health but also a key strategy for the prevention of cardiovascular diseases.

There are various methods to improve aerobic fitness, such as swimming, aerobics, and cycling [14]. However, running stands out as the most cost-effective and accessible form of aerobic exercise [15]. The positive effects of running on enhancing physical fitness have been widely confirmed. Studies have shown that both high-intensity interval training and continuous endurance training can significantly enhance individual aerobic fitness and improve body composition [16–18]. Fun Running, as a low-intensity form of endurance training, has become a popular activity for college students [19]. It not only demonstrates significant effectiveness in enhancing aerobic fitness but also plays a pivotal role in cultivating a positive sports culture on campus [20]. These findings provide a scientific basis for the diversification of training for running, confirming the positive contribution of various running styles to health. However, it is worth noting that there is a dose-response relationship between different forms of running and health outcomes in aerobic fitness.

The dose-response relationship refers to the physiological response to a given training load [21]. It should be noted that an individual’s exercise tolerance may vary due to their biological adaptability to exercise, which ultimately determines the appropriate and effective exercise dose [22]. Therefore, a core issue in developing effective exercise prescriptions is to understand the impact of different physical activity doses on health and health outcomes through empirical evaluation and comparison of actual doses. There is a wealth of studies about aerobic fitness training and loads, as well as many studies documenting dose-response. The World Health Organization has recommended that individuals engage in 150 min of moderate physical activity or 75 min of vigorous-intensity aerobic exercise weekly to attain longevity benefits [23]. Several other studies [24, 25] have also established different levels of physical activity based on the extent of sedentary behavior, considering varying intensities, durations, and frequencies.

However, the debate is ongoing about what exercise loads lead to better aerobic fitness and the lower limits of these loads. Moreover, the evidence regarding dose-response relationships between exercise load and outcomes in aerobic fitness in college students remains very limited. To the best of our knowledge, there are few studies have focused on the dose-response relationship between running and aerobic fitness among college students in the post-epidemic era. Therefore, this study aimed to investigate the dose-response relationship between 15 weeks of running and eaerobic fitness based on the Fun Running program of Wannan Medical College. The purpose of our study is (1) To provide deep insight into the impact of the Fun Running program on aerobic fitness development among college students. (2) To examine the dose-response relationship between the components of Fun Running and aerobic fitness and formulate a more effective Fun Running program.

## Method

### Participants and ethics

The basic information of the participants is presented in Table 1. A total of 3224 college students (1694 females, 1530 males) enrolled at Wannan Medical College in 2022 were involved. The average age of participants is 18.5 ± 0.6 years, and the average BMI is 21.8 ± 3.5 kg/m^2^. To ensure that the results of Fun Running programs accurately reflected the natural aerobic fitness levels of the participants, we did not implement any additional physical training for the participants beyond regular physical education classes. Meanwhile, to safeguard the aerobic fitness test and the Fun Running program, participants with health conditions requesting exemption from the physical fitness test were excluded (32 females, 34 males). The study was approved by the Ethics Review Committee of Wannan Medical College, and the consent of all participants was obtained.

**Table 1:** The basic information of the participants (Mean ± SD)

	Total(*n* = 3224)	Female(*n* = 1694)	Male(*n* = 1530)
Age(years)	18.5 ± 0.6	18.6 ± 0.8	18.4 ± 0.4
BMI(kg/m^2^)	21.8 ± 3.5	21.1 ± 3.1	22.6 ± 3.8

### Study design

This is a retrospective study conducted by reviewing the Fun Running program at Wannan Medical College in China. A pre-post study design was performed by comparing the changes in MLDR-P before and after a 15-week Fun Running program that was implemented from February to June of 2023. We compared the differences between the two MLDR-Ps and examined the dose-response relationship between the three components of the Fun Running and MLDR-P.

### Fun running program and measurement

The Fun Running application (version 3.8.0, Fun Running Sports Internet Co., Ltd., Wuhan) serves as a mobile phone-based tool for recording various data related to Fun Running. This includes information on frequency, pace, and distance. To adhere to the program, certain rules were put in place: (1) The distance covered during each run must meet the standard, with a minimum of 2.5 km for female students and 3.0 km for female students. (2) The pace of each run should range between 3 min/km and 9 min/km. (3) The running route of each run must pass through three designated check-in locations, which are randomly assigned by the app. To encourage students to participate in the Fun Running program, the school stipulates that students can receive 1 point for each Fun Running, with a maximum limit of 30 points, which will be included in the final physical education score (30%).

The measurement of Fun Running involves a series of steps. First, participants are required to initiate the app on their mobile phones before commencing their run. They must grant permission for GPS positioning to be used by their devices. Second, participants are required to press the “complete” button and upload the results at the end of each run. Last, the app automatically evaluates the pace and distance achieved during the run and compares it with the three rules of the Fun Running program, only runs that adhere to these rules will be included in the final results. All Fun Running component data were obtained from the supplier at the end of the Fun Running program.

### Aerobic fitness and measurement

According to the Chinese “National Physical Fitness Test Standards”, for college students, the performance of 800-meter running for females and 1000-meter running for males is considered the primary indicator of aerobic fitness [26]. In this study, we collectively refer to these distances as middle and long-distance running.

At Wannan Medical College, students are required to complete the middle and long-distance running test at the end of each semester unless they have applied for an exemption. It was counted as part of their physical education course grade (10%). For this study, we selected the results of two middle and long-distance running tests, conducted before and after the Fun Running program. Both tests were administered by the same group of physical education teachers at Wannan Medical College. The performance of the 800/1000 meter run was recorded using a stopwatch (YS-810, Yisheng Technology Co., Ltd., Shenzhen, China) and measured in minutes. All middle and long-distance running test results were obtained from the Department of Physical Education of Wannan Medical College. In our study, MLDR-P was used as a measure of aerobic fitness. It was calculated using the following formula:

MLDR-*P* = Duration (min)/Distance (km).

### Statistical analysis

SPSS 27.0(SPSS Inc.) was used for statistical analysis. Descriptive statistics are presented as means and standard deviations (Mean ± SD). Standardized residual plots (histograms, normal probability plots), Durbin-Watson tests, and variability measures were used to examine the variable distributions. A paired samples T test was used to analyze the differences between the two MLDR-Ps, with the effect size (Cohen’s d). The magnitude of change was interpreted based on the following criteria: > 0.2–0.6, small; > 0.6–1.2, moderate; > 1.2, large [27]. Pearson’s correlation coefficient was used to examine the strength and direction of the dose-response relationship between the Fun Running components and MLDR-P. The magnitude of the relationship was interpreted using the following criteria: < 0.3 low; > 0.3–0.5 moderate; > 0.5–0.7 high; > 0.7 very high [27]. Multiple regression analysis was used to examine the association between the components of Fun Running and MLDR-P. The coefficient of determination (R^2^) was calculated via linear regression analysis to determine the level of variance in MLDR-P explained by Fun Running. Standardized regression coefficients were also calculated to understand the extent to which each component of the Fun Running affected the MLDR-P. Each dependent variable was analyzed separately, with the models adjusted for sex. A significance level of *P* < 0.05 (2-tailed) was considered indicative of statistical significance.

## Results

Table 2 shows the results of the three components of Fun Running. For females, the mean and standard deviation for frequency were 30.5 ± 2.5, for distance were 2.20 ± 0.19 km, and for pace were 7.01 ± 0.72 min/km. For males, the corresponding values were 30.3 ± 4.1, 3.07 ± 0.29 km, and 6.30 ± 0.76 min/km, respectively.

**Table 2:** Description of three components of fun running (Mean ± SD)

	Female(*n* = 1694)	Male(*n* = 1530)
Frequency(time)	30.5 ± 2.5	30.3 ± 4.1
Distance(km)	2.20 ± 0.19	3.07 ± 0.29
Pace(min/km)	7.01 ± 0.72	6.30 ± 0.76

Table 3 shows the results of the comparison of MLDR-P before and after the Fun Running program. The changes in MLDR-P were found to be significant for both female and male students. Specifically, the mean MLDR-P improved by 0.11 (*P* < 0.001, d = 0.68) for females and 0.09 (*P* < 0.001, d = 0.72) for males.

**Table 3:** Comparison of MLDR-P before and after the fun running program (Mean ± SD)

	Before	After	Change	*P*	Cohen’s d
MLDR-P-F^1^(min/km)	4.95 ± 0.44	4.84 ± 0.53	0.11 ± 0.68	< 0.001	0.68
MLDR-P-M^2^(min/km)	4.19 ± 0.45	4.10 ± 0.52	0.09 ± 0.72	< 0.001	0.72

Notes: ^1^Middle and long-distance running pace of female students; ^2^Middle and long-distance running pace of male students; The same is below

Table 4 shows the correlation between MLDR-P and the three components of the Fun Running. All three components of Fun Running displayed significant intercorrelations with MLDR-P. Specifically, MLDR-P-F showed a high positive correlation with pace (*r* = 0.95) and a high negative correlation with distance (*r* = -0.78), while showing the lowest correlation with frequency (*r* = -0.22). Similar results were apparent with MLDR-P-M as the pace (*r* = 0.98), distance (*r* = -0.67), and frequency (*r* = -0.18). Furthermore, it is worth noting that pace shows a high negative correlation with distance (*r* = -0.90, for females, *r* = -0.75, for males).

**Table 4:** Correlation between MLDR-P and fun running components

	Frequency(N)		Distance(km)		Pace(min/km)	
	r	95% CI	r	95% CI	r	95% CI
MLDR-P-F(min/km)	-0.22^※^	-0.26, -0.17	**-0.78** ^※^	-0.80, -0.76	**0.95** ^※^	0.95, 0.96
Frequency(N)			0.46^※^	0.42, 0.50	-0.30^※^	-0.34, -0.25
Distance(km)					**-0.90** ^※^	-0.91, -0.90
MLDR-P-M(min/km)	-0.18^※^	-0.23, -0.13	**-0.67** ^※^	-0.70, -0.64	**0.98** ^※^	0.97, 0.98
Frequency(N)			0.45^※^	0.41, 0.49	-0.21^※^	-0.25, -0.16
Distance(km)					**-0.75** ^※^	-0.77, -0.72

Notes: ^※^Indicates statistical significance (*P* < 0.05); Bold values mean high correlation

Table 5 shows the results of the statistical analysis of the regression model with MLDR-P as the dependent variable. All three components of Fun Running can significantly explain the MLDR-P, be it MLDR-P-F (R^2^ = 0.95, *p* < 0.05) or MLDR-P-M (R^2^ = 0.96, *p* < 0.05). Amongthe components, pace demonstrated the greatest impact on MLDR-P (β = 1.39, for females; β = 1.09, for males), followed by distance (β = 0.49, for females; β = 0.15, for males), and frequency demonstrates the smallest impact on MLDR-P (β = -0.03, for all).

**Table 5:** Regression analysis of MLDR-P and fun running components

Model		B	B’ 95% CI	β	*P*	R^2^
MLDR-P-F	Intercept	-5.278	-5.569, -4.986		< 0.001	0.95
	Frequency(N)	-0.007	-0.010, -0.004	-0.03	< 0.001	
	Distance(km)	1.398	1.315, 1.481	0.49	< 0.001	
	Pace(min/km)	1.034	1.014, 1.054	1.39	< 0.000	
MLDR-P-M	Intercept	-1.295	-1.428, -1.162		< 0.001	0.96
	Frequency(N)	-0.003	-0.005, -0.002	-0.03	< 0.001	
	Distance(km)	0.267	0.238, 0.296	0.15	< 0.000	
	Pace(min/km)	0.741	0.731, 0.751	1.09	< 0.001	

**Fig. 1:**
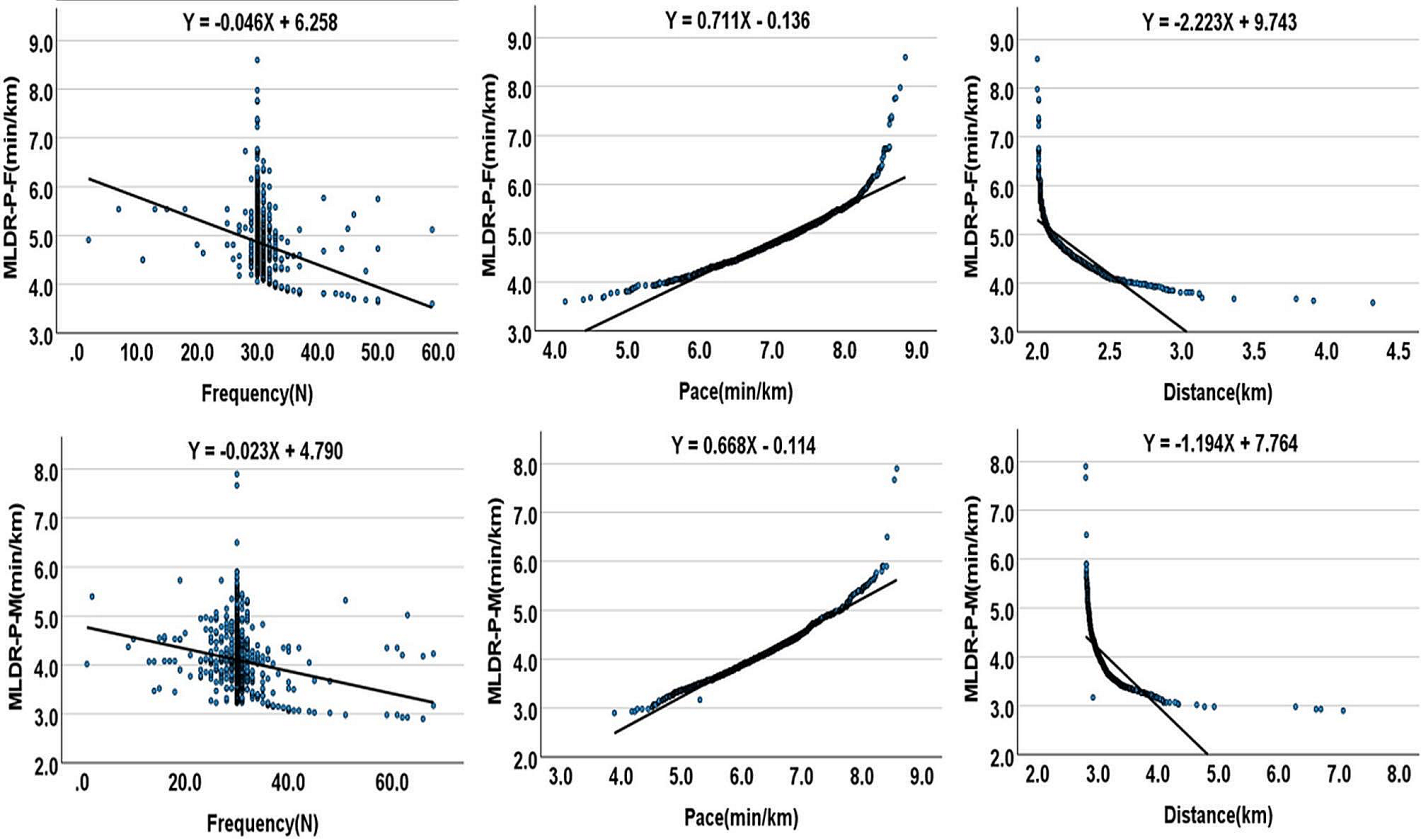
Regression equation between fun running components and MLDR-P

Table 6 shows the minimum frequency, pace, and distance needed for each level of MLDR-P. According to the Chinese “Physical Health Test Standards for College Students“ [26] and regression equation (Fig. 1), we calculated the minimum training load needed to elicit a change in each level of middle and long-distance running performance.

**Table 6:** The minimum training load for each level of middle and long-distance running

		MTL to elicit MLDR-P-L^1^		
	Level Peac(min/km)	Frequency(N)	Pace(min/km)	Distance(km)
MLDR-P-F	Excellent(pace ≤ 4.38 )	41	6.35	2.4
	Good(4.38 < pace ≤ 4.67 )	35	6.76	2.3
	Passing(4.67 < pace ≤ 5.71)	12	8.22	1.8
MLDR-P-M	Excellent( peac ≤ 3.45 )	58	5.34	3.6
	Good(3.45 < peac ≤ 3.70 )	47	5.71	3.4
	Passing(3.70 < peac ≤ 4.53)	11	6.95	2.7

Notes: ^1^The minimum training load required to elicit the middle and long-distance running performance levels change

## Discussion

This study fills the gap in research on the dose-response relationship between running and aerobic fitness among college students in the post-epidemic era. A pre-post study design was performed by comparing the changes in MLDR-P before and after a 15-week Fun Running program. The key finding was that 15-week Fun Running can significantly improve the MLDR-P. Furthermore, the pace of Fun Running had the highest correlation with changes in MLDR-P, followed by the distance covered, while frequency displayed the weakest correlation.

Previous studies have shown that long-term regular aerobic exercise, such as swimming, cycling and running can significantly improve aerobic endurance [28–32]. Long-term regular swimming is effective in improving aerobic fitness [28, 29], and as a low-impact exercise, it is particularly popular with obese people, the elderly, and people with arthritis because it does not involve weight-bearing [33]. Similarly, cycling has shown a positive correlation with aerobic fitness, and it can not only improve aerobic fitness but also promote overall health [30–32]. Consistent with these results, our study indicated that a 15-week Fun Running program had a significant positive impact on the MLDR-P of college students. Long-term aerobic activities can enhance the body’s capacity to inhale, convey, and utilize oxygen [34], as well as improve the body’s tolerance to lactic acid [35], thus promoting exercise continuity and improving efficiency [18]. Exercise performance improves along with improvements in exercise efficiency [36]. In our study, the MLDR-P was decreased by 0.11 min/km for females and 0.09 min/km for males following the Fun Running program, which implies that female individuals saved 6.8 s and male individuals saved 6.6 s in their middle and long-distance running performance. These findings suggest that the Fun Running program can be used as a means to improve the aerobic fitness of college students in the post-pandemic era.

We also found that all three components of the Fun Running are significantly related to the MLDR-P and can significantly explain it. This is not the same as previous research suggesting that benefits can be achieved by modifying any of the components of exercise [37–39]. Our results revealed that pace exhibited the highest correlation with changes in MLDR-P, followed by distance, and frequency had the lowest correlation. More importantly, we observed a highly negative correlation between the pace of Fun Running and distance, meaning that engaging in long-distance running may decrease the pace, which may not be conducive to improving the MLDR-P. By comparing the standardized regression coefficients obtained from our regression analysis, we found that for every unit increase in pace, distance, and frequency, the MLDR-P improved by 1.39, 0.49, 0.03 for females and 1.09, 0.15, 0.15 for males. These results suggest that when seeking to develop an effective Fun Running program, focus should be placed on pace, although we acknowledge that more distance and frequency may also be advantageous.

The results also indicated that the minimum Fun Running load needed to elicit a change in middle and long-distance running performance levels does not align with the established rules of the Fun Running program. These results suggest that a hierarchical set of guidelines for the Fun Running program should be developed, taking into account the varying levels of middle and long-distance running achievements among students. This approach will help enhance the aerobic fitness of all students.

This study presents a comprehensive overview of the practical application of aerobic fitness training methods in college during the post-epidemic era. This study is important for improving the theoretical framework of physical training for students. Moreover, it also has important practical significance for the recovery of students’ physical fitness following public health incidents.

There are two main strengths in this study. (1) The sample size of the study is 3244 college students, which is larger than that of previous studies, making the data more powerful and the results more reliable. (2) Our study has established clear guidelines for the Fun Running program designed specifically for Chinese college students, which will help participants develop effective prescriptions. However, there are some limitations in this study. (1) Limitations of the geographic distribution of the sample. By focusing only on specific regions, we failed to capture the variability in the impact of the COVID-19 outbreak on different regions, which may have affected the broad applicability of the assessment of the effects of the Le Running program. (2) Limitations in the selection of the study population. We focused on the medical college student population and failed to include a broader population of different age groups and professional backgrounds, which limits the general applicability of the findings. (3) In analyzing factors contributing to changes in MLDR-P, our study focused primarily on the Fun Running program while assuming that students did not engage in additional physical activity beyond the program and required physical education classes. This assumption ignored other confounding variables that may have an impact on aerobic fitness, such as an individual’s diet, lifestyle habits, and exercise habits. Future studies should be expanded to multiple regions, involve diverse populations, and use a variety of methods including questionnaires and health monitoring in order to more accurately assess the effects of the Fun Running program on aerobic fitness.

## Conclusion

This study aims to provide deep insight into the impact of Fun Running on the aerobic fitness development of college students. Results show that 15 weeks of Fun Running can significantly improve aerobic fitness. Examination of the dose-response relationship between Fun Running and MLDR-P provides practitioners with valuable insights into prescribing aerobic fitness training, allowing them to develop more effective training programs. In the future, how to effectively implement the Fun Running program, which is based on hierarchical rules, is worthy of study regarding the recovery of aerobic fitness for all students.

## Data Availability

The datasets used and/or analyzed during the current study are available from the corresponding author upon reasonable request.

## Abbreviations

MLDR-P: Middle and long-distance running pace
MLDR-P-F: Middle and long-distance running pace of female students
MLDR-P-M: Middle and long-distance running pace of male students
P: Sign value
ES: Effect size
Mean ± SD: Means and standard deviations
r: Pearson correlation coefficient
B: Unstandardized regression coefficients
β: Standardized regression coefficient
R^2^: Determination coefficient
MTL to elicit MLDRPL: The minimum training load required to elicit the middle and long-distance running performance levels change
